# Prediction of Carbohydrate-Binding Proteins from Sequences Using Support Vector Machines

**DOI:** 10.1155/2010/289301

**Published:** 2010-09-27

**Authors:** Seizi Someya, Masanori Kakuta, Mizuki Morita, Kazuya Sumikoshi, Wei Cao, Zhenyi Ge, Osamu Hirose, Shugo Nakamura, Tohru Terada, Kentaro Shimizu

**Affiliations:** ^1^Department of Biotechnology, The University of Tokyo, 1-1-1 Yayoi, Bunkyo-ku, Tokyo 113-8657, Japan; ^2^Agricultural Bioinformatics Research Unit, The University of Tokyo, 1-1-1 Yayoi, Bunkyo-ku, Tokyo 113-8657, Japan

## Abstract

Carbohydrate-binding proteins are proteins that can interact with sugar chains but do not modify them. They are involved in many physiological functions, and we have developed a method for predicting them from their amino acid sequences. Our method is based on support vector machines (SVMs). We first clarified the definition of carbohydrate-binding proteins and then constructed positive and negative datasets with which the SVMs were trained. By applying the leave-one-out test to these datasets, our method delivered 0.92 of the area under the receiver operating characteristic (ROC) curve. We also examined two amino acid grouping methods that enable effective learning of sequence patterns and evaluated the performance of these methods. When we applied our method in combination with the homology-based prediction method to the annotated human genome database, H-invDB, we found that the true positive rate of prediction was improved.

## 1. Introduction

Sugar chains and carbohydrate-binding proteins play important roles in several biological processes such as cell-to-cell signaling, protein folding, subcellular localization, ligand recognition, and developmental processes [1]. With the rapid increase in the amount of available glycoprotein data (i.e., protein sequences), there is a growing interest in the functions, physicochemical properties, and tertiary structures of carbohydrate-binding proteins and in their applications. Experimental work to identify carbohydrate-binding proteins is costly and time consuming, so computational methods to predict carbohydrate-binding proteins would be useful.

Carbohydrate-binding proteins are nonantibody proteins that can interact with sugar chains, and various keywords are used to annotate them in biological databases: “carbohydrate-binding protein”, “lectin”, and so on. The term “lectin” is widely used but there is no general consensus as to its definition. The Shiga toxin B subunit, for example, has been annotated “lectin-like” as well as “lectin.” Furthermore, heparin-binding proteins and hyaluronic-acid-binding proteins are also carbohydrate-binding proteins, but are not usually annotated “carbohydrate-binding protein” or “lectin.” In the work reported in this paper, we first collected carbohydrate-binding proteins of various kinds, including enzymes and proteins not explicitly annotated as “carbohydrate-binding protein,” and specified a set of search conditions for carbohydrate-binding proteins in the amino acid sequence database UniProt Knowledgebase (UniProtKB). Based on the collected proteins, we developed a carbohydrate-binding protein prediction system by using machine learning methods, with which predicting carbohydrate-binding proteins can be formulated as a binary classification problem.

We used support vector machines (SVMs) [2] to create a classifier to predict whether a target protein is a carbohydrate-binding protein. SVMs are supervised learning algorithms used for binary classification problems, and they can handle noisy data and high-dimensional feature spaces. They therefore often perform well in classification problems such as protein secondary structure prediction [3], disorder prediction [4], and fold recognition [5]. To the best of our knowledge, however, the only reported methods for predicting carbohydrate-binding proteins are conventional homology-based methods. Although methods predicting carbohydrate-binding sites by using empirical rules [6] or a machine learning method [7] have been developed and could in principle be used to predict carbohydrate-binding proteins, for example, by using the maximum scores of possible binding sites, they are not designed to predict negative instances (noncarbohydrate-binding proteins); they are designed for predicting binding sites of proteins that are already known as carbohydrate-binding proteins. Furthermore, they generally need much computation time and often require three-dimensional protein structure data. Our SVM-based method uses only sequence information and can be applied to many proteins whose structures are not determined. It also requires less computation time and can be used for genome-wide analysis.

The encoding of the sequences for feature extraction is an important factor affecting the ability of SVMs to discriminate sequences and amount of computation time required for that discrimination. In this study we assessed two kinds of encoding methods: direct encoding and group encoding. In the direct encoding method, the features of the amino acid sequences were represented by triplets of amino acid patterns. In the group encoding method, twenty amino acids were first grouped according to their properties and then the features of amino acid sequences by using frequencies of triplets of the group symbols. In both kinds of methods, we used a 3-spectrum kernel [8] because it is a conceptually simple and computationally efficient kernel for string of symbols.

## 2. Material and Methods

**Figure 1:**
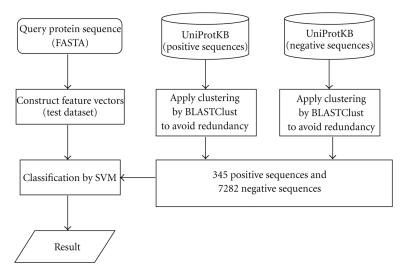
Outline of prediction system.

Figure 1 shows an outline of our SVM-based prediction system. We constructed the positive and negative datasets with which the SVMs were trained.

### 2.1. Construction of Positive and Negative Datasets

To construct the positive dataset, we defined carbohydrate-binding proteins as proteins, other than antibodies, that can interact with sugar chains but cannot modify them [1]. 

The sequences of carbohydrate-binding protein sequences were collected from UniProtKB [9] by using a sequence retrieval system (SRS) as follows. We first constructed intermediate datasets by writing commands in SRS query language commands specifying the protein-retrieving condition (Table 1). The sequences were extracted from UniProtKB when the conditions matched the annotation of proteins. We determined the contents of the commands, according to the following references: [10–21]. Although the sequences extracted were not those of all carbohydrate-binding proteins, we intended to collect a wide range of carbohydrate-binding proteins based on published papers. We merged the intermediate datasets, which were retrieved from UniProtKB with the conditions listed in Table 1, and then removed redundancy between the sequences, yielding the positive dataset containing 345 carbohydrate-binding proteins sequences.

Note that the intermediate datasets contained no proteins annotated “Putative” in “DE” (description) lines of their UniProtKB entries. That annotation is based only on sequence similarities and with little experimental evidence, so they might not actually be carbohydrate-binding proteins [22, 23]. In addition, the proteins in the intermediate datasets have more than 30 amino acids and are not inferred proteins.

To remove the sequence redundancy of the positive dataset, we first clustered sequences with BLASTClust [24, 25]. We put the sequences into the same cluster if the sequence identity exceeded 35% in at least one sliding window whose width was 40% of the sequence length. Then for each sequence we calculated the sum of evolutionary distances from all other sequences in the same cluster. The sequence with the smallest sum of distances was selected as the representative sequence for that cluster. The evolutionary distance between two sequences was calculated from pairwise scores for the sequences by using ClustalW [26].

A sequence-based conserved domain search against NCBI Conserved Domains Database (CDD) [27] v.2.21 found 273 proteins (79.1%) with *E* value <10^−2^ and 249 proteins (72.2%) with *E* value <10^−5^ in the positive dataset to have one or more carbohydrate-binding domains. The numbers of carbohydrate-binding domains found in the 345 proteins of the positive dataset were 149 with *E* value <10^−2^ and 143 with *E* value <10^−5^. Since there are 185 carbohydrate-binding domains in the CDD, the positive dataset contains about 80% of them. These carbohydrate-binding domains include several kinds of C-type lectin domains, galactose- or galactoside-binding lectin domains, chitin-binding lectin domains, and so on. They are summarized in Table S1 in supplementary material available online at doi:10.1155/2010/289301.

The sequences for the negative dataset were randomly collected from UniProtKB [9] using the same number of sequences in the positive dataset under the following conditions: (1) proteins in the dataset must not match any sequence in the positive dataset; (2) the entries must not be annotated as “Putative” in “DE” (description) lines of their UniProtKB entries; (3) the proteins must have more than 30 amino acids; (4) the proteins are not inferred proteins. The sequence redundancy of the negative dataset was removed using the same clustering and selection algorithms that were used for the positive dataset.

### 2.2. SVM Training and Prediction

An SVM maps feature vectors into a high-dimensional space and classifies samples by setting the hyperplane in this space. During training, an SVM defines an optimal hyperplane maximizing the margin between two classes. A maximum-margin hyperplane has good generalization performance.

To discriminate carbohydrate-binding proteins from noncarbohydrate-binding proteins, we trained the SVM with three different encoding methods: a direct encoding method (AA-20), and two grouping methods (Levitt-6 and Someya-7). In AA-20, triplets of amino acid patterns are used to represent the features of the amino acid sequences as described later. In Levitt-6, twenty amino acids are assigned to six residue groups, and the amino acid sequences are converted into sequences of the six group symbols. This method uses as features of the sequences the frequencies of triplets of group symbols.

The Levitt-6 grouping method (described in Table 2(a)) is based on the polarity and the propensity of the secondary structure of each amino acid [28]. It is well known that around the carbohydrate-binding interface there are hydrogen bonds and a stacked arrangement of aromatic amino acids between polar amino acids and the ligand (carbohydrate) [29]. Carbohydrate-binding proteins also have a variety of structures consisting of *β* folds [1, 30–33], the *β* barrel (i.e., jelly roll), and the *β* trefoil. We therefore expected Levitt-6 to be an effective means of extracting sequence features.

As described in Section 3, there is an extremely large difference between the cysteine frequencies of carbohydrate-binding proteins and those of other proteins. Cysteine brings amino acids that are distant in the primary structure or are in different polypeptides close to each other through a disulfide bond, and some families of carbohydrate-binding proteins contain a cysteine-rich domain [1, 34] Therefore, in the Someya-7 grouping method we treat cysteine as a separate group (see Table 2(b)); twenty amino acids are categorized into seven groups and the amino acid sequences are converted into sequences of the seven group symbols. This method also uses the frequencies of triplets of the group symbols as sequence features.

We used LIBSVM (http://www.csie.ntu.edu.tw/~cjlin/libsvm) as the SVM implementation. As an SVM kernel, we used a Gaussian kernel, one of the radial basis function (RBF) kernels that enable an SVM to handle nonlinear classification. The SVM with a Gaussian kernel has two parameters: gamma and cost (*C*). Gamma determines the Gaussian kernel function, and *C* determines the hyperplane softness. Parameter *C* and gamma are optimized by using grid.py script, which is included in the LIBSVM package.

SVM decision values were used for the classification. If the value was higher than or equal to a specified threshold, the corresponding protein was predicted to be a carbohydrate-binding protein. If a decision value was below the threshold, the corresponding protein was predicted to be a noncarbohydrate-binding protein. We used spectrum kernel [8] that has been used to solve many sequence classification problems. A sequence is considered a string of finite set of twenty characters. The kernel can be briefly described as follows.

Let A denote a set of finite symbols (i.e., a single letter code of 20 amino acids or a group symbol of amino acids in our system), and let *x* and *y* denote two strings defined on alphabet A. The *k*-spectrum kernel is then defined as follows [8].

First, we define *ϕ*_*α*_(*x*), which represents the number of occurrences of the *k*-length substring *α* in the sequence *x*, as follows:

(1) $$\Phi_{k}={\left(\phi_{\alpha }\left(x\right)\right)}_{\alpha \in {\text{A}}^{k}}.$$

If *x* and *y* share many of the same *k*-length substrings, the two strings are considered similar. The *k*-spectrum kernel of *x* and *y* is obtained by taking the dot product of the corresponding *k* spectra:

(2) $${\text{K}}_{k}^{\text{spct}}\left(x,y\right)=\langle \Phi_{k}\left(x\right),\Phi_{k}\left(y\right)\rangle .$$

In this study, we used a normalized kernel defined as follows:

(3) $${\text{K}}_{k,\mathrm{norm}\;}^{\text{spct}}\left(x,y\right)=\frac{{\text{K}}_{k}^{\text{spct}}\left(x,y\right)\;}{\sqrt{{\text{K}}_{k}^{\text{spct}}\left(x,x\right)}\sqrt{{\text{K}}_{k}^{\text{spct}}\left(y,y\right)}}.$$

We set *k* = 3. In this experiment, the number of dimensions of the input space was 8000 ( = 20^3^) in AA-20, 216 ( = 6^3^) in Levitt-6, and 343 ( = 7^3^) in Someya-7.

Our Gaussian kernel was constructed by using the spectrum kernel as follows:

(4) $$\begin{array}{cc} \text{K}\left(x,y\right) & =\exp\;\;[-\gamma \{{\text{K}}_{k,\mathrm{norm}\;}^{\text{spct}}\left(x,x\right)-2{\text{K}}_{k,\mathrm{norm}\;}^{\text{spct}}\left(x,y\right)\\ & \;\;\;\;\;+{\text{K}}_{k,\mathrm{norm}\;}^{\text{spct}}\left(y,y\right)\}], \end{array}$$

which was used for classification.

### 2.3. Performance Measurement

We assessed the discrimination ability of our prediction system by using the following measures: accuracy (ACC), true positive rate (TPR), false positive rate (FPR), and the Matthews correlation coefficient (MCC). These measures were calculated as follows:

(5) $$\begin{array}{c} \text{Accuracy}=\frac{\text{TP}+\text{TN}}{\text{TP}+\text{TN}+\text{FP}+\text{FN}}\times 100,\\ \text{MCC}=\frac{\text{TP}\times \text{TN}-\text{FP}\times \text{FN}}{\sqrt{\left(\text{TP}+\text{FN}\right)\left(\text{TP}+\text{FP}\right)\left(\text{TN}+\text{FP}\right)\left(\text{TN}+\text{FN}\right)}},\\ \;\text{True}\;\text{positive}\;\text{rate}\;\left(\text{TPR}\right)=\frac{\text{TP}}{\text{TP}+\text{FN}}\times 100,\\ \;\text{False}\;\text{positive}\;\text{rate}\;\left(\text{FPR}\right)=\frac{\text{FP}}{\text{FP}+\text{TN}}\times 100. \end{array}$$

Here TP, FN, FP, and TN, respectively, represent the numbers of true positives, false negatives, false positives, and true negatives.

The ROC curve [35] is a two-dimensional graph in which TPR is plotted against FPR. Therefore, each classification threshold value *θ*, which corresponds to particular values of TPR and FPR, produces a different point on an ROC curve. ROC curves depict the tradeoff between true positive and false positive.

To evaluate how well classifiers discriminate between positive and negative instances, we also calculated the area under the ROC curve (AUC), which represents the probability of correct classification, with an AUC of 0.5 indicating a random discrimination between positives and negatives (a random classifier) and an AUC of 1 indicating perfect discrimination.

The performance measures described above were evaluated by the leave-one-out method. This method is a type of cross-validation test where a dataset consisting of *N* sequences is divided into *N* subsets. The classifier was trained on *N*−1 subsets and tested with a subset not used for training. The process was repeated *N* times, using each subset as the test set and the rest of the subsets as the training set. In the leave-one-out test we optimized the SVM parameters gamma and cost (*C*) with each subtraining set.

### 2.4. Log-Odds Ratios of Amino Acid Frequencies

The log-odds ratio of the frequency of amino acid type *i* between the positive dataset and the background was calculated as

(6) $$R_{i}={\log\;}_{2}\;\frac{f_{p,i}}{f_{s,i}},$$

where *f*_*p*,*i*_ represents the observed frequency of amino acid type *i* in the positive dataset (*p*) and *f*_*s*,*i*_ represents the background frequency.

The observed frequency of amino acid type *i* of the positive dataset is given by

(7) $$f_{p,i}=\frac{\sum_{j\in p}n_{j,i}}{\sum_{i}\sum_{j\in p}n_{j,i}},$$

where *j* represents the sequence in the dataset.

The background frequencies of 20 amino acids were obtained from the Swiss-Prot protein knowledgebase release 56.4 statistics (http://br.expasy.org/sprot/relnotes/relstat.html).

### 2.5. Homology-Based Prediction

Our homology-based prediction method used the 345 sequence clusters of carbohydrate-binding proteins from which the positive dataset was constructed as described in Section 2.1. The sequence homology search was applied to these sequence clusters as follows.

1. For clusters consisting of a single sequence (167 clusters), a query sequence was compared with the sequence by BLAST.
2. For clusters consisting of multiple sequences (178 clusters), a position-specific scoring matrix (PSSM) was constructed from the sequences in each cluster and a query sequence was compared with the PSSM by RPS-BLAST [27].

We judged a query sequence to be a carbohydrate-binding protein when the *E* value of the BLAST/RPS-BLAST search was less than a specified value (currently 10^−10^). We combined the homology-based method and the SVM-based method. In the combined prediction, Someya-7 was used as the SVM sequence encoding method. We applied the combined method to H-Invitational Database (H-InvDB) [36].

## 3. Results

One of the main results of this study is that the SVM classifier consistently showed an ability to correctly discriminate between carbohydrate-binding proteins and noncarbohydrate-binding proteins. The classifier only requires the sequence of each protein. 

**Figure 2:**
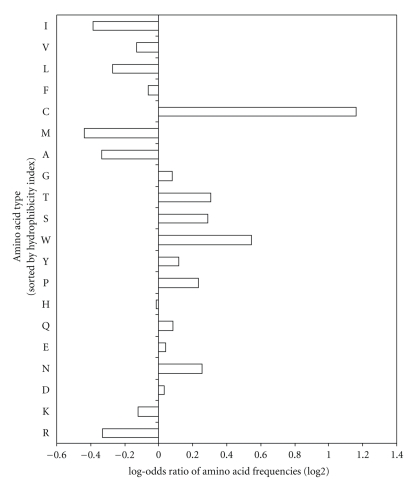
Amino acid frequencies of carbohydrate-binding proteins.

We calculated log-odds ratios of 20 amino acid frequencies of the positive dataset and the background frequencies obtained from Swiss-Prot. The result of this calculation (Figure 2) shows the characteristic distribution of amino acids in carbohydrate-binding proteins, which is used for learning of our prediction system. We also based the amino acid grouping methods (described in Section 2.2) on this distribution of amino acids.

The performance of our prediction system was assessed by using the leave-one-out method and the following measures: accuracy (ACC), true positive rate (TPR), false positive rate (FPR), and Matthews correlation coefficient (MCC). The ACC, TPR, FPR, and MCC vary with the classification thresholds (decision value), while the AUC is independent of the threshold. Therefore, we mainly used AUC for performance evaluation.

In the direct encoding method (here denoted *AA-20*), twenty kinds of amino acids were used to represent sequence patterns directly. As group encoding methods we used the amino acid grouping proposed by Levitt [11] (here denoted *Levitt-6*) and a modification of it (proposed in this paper and here denoted *Someya-7*).

**Figure 3:**
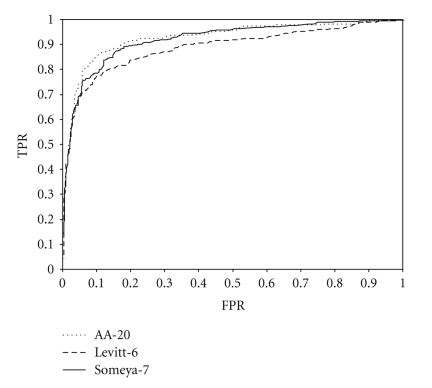
Prediction performance.

Figure 3 shows the ROC curve for each method. For most false positive rate values, Someya-7 shows a true positive rate almost equal to that of AA-20. The performance of each classifier is shown by the value listed in Table 3. The AUC values for Someya-7 and AA-20 were, respectively, 0.918 and 0.929; almost equal to each other and higher than the AUC for Levitt-6 (0.890).

We examined a homology-based search in which a query sequence is compared by BLAST. The most similar protein in the dataset (excluding the query sequence) was searched and when it is a positive (negative) sequence the query sequence is predicted as a carbohydrate-binding (noncarbohydrate-binding) protein. The AUC value for this method is 0.848 which was lower than our SVM-based method. 

Because it is very difficult to evaluate the performance of the homology-based method when using a small test dataset, we used it on 185,003 sequences of H-Invitational proteins (HIPs) (Release 5.0) in H-InvDB [36]. Since H-invDB is not fully annotated, we regarded a protein sequence as that of a carbohydrate-binding protein (positive) if it had a sequence identity higher than 98% to any sequence annotated with one of the keywords listed in Table 1. In this evaluation, we implemented a homology-based prediction method in which a query sequence is compared by BLAST or RPS-BLAST [27] with known carbohydrate-binding proteins. (The method is described in detail in Section 2.5.) This method was also combined with the SVM-based method.

**Figure 4:**
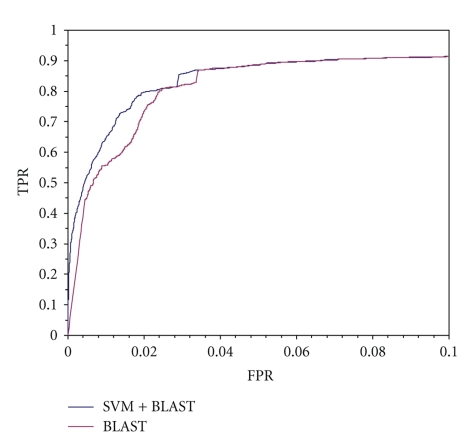
Performance in genome-wide prediction.

Figure 4 shows ROC curves for the homology-based method and the combined method, in which a sequence is predicted as positive only when both the SVM-based method and the homology-based method predict it as positive. The combined method shows higher TPR values than the homology-based method especially when FPR value is low. (The performance at low FPR values is important in practice.) When the FPR was 0.015, the combined method predicted 3,810 sequences as positive whereas the homology-based method predicted 5,523 sequences as positive which contain more false positives. As shown in Figure 4, the TPR was improved from 0.639 to 0.727. The SVM-based method itself predicted that only 492 sequences would be those of carbohydrate-binding proteins.

## 4. Discussion

The AUCs of AA-20, Levitt-6, and Someya-7 were, respectively, 0.929, 0.890, and 0.918. These values show that our prediction system successfully discriminated carbohydrate-binding proteins from other proteins.

We used a 3-spectrum kernel for each grouping method. In AA-20 the number of dimensions of the input space was large (8000 = 20^3^) compared to the number of training samples (766). In general, if there are too few training samples relative to the dimensions of the space, an inappropriate prediction model might be constructed by overfitting to the training data. We therefore also used the Levitt-6 and Someya-7 grouping methods, in which the numbers of dimensions of the input spaces were, respectively, 216 (= 6^3^) and 343 (= 7^3^), both much smaller than 8000 (= 20^3^). We expected that using these methods would help the SVMs avoid overfitting problems.

The choice of grouping criteria directly affects the SVMs discrimination ability. If the choice is inappropriate, the features of carbohydrate-binding proteins will not be detected, and performance will deteriorate even if the size of the input space is appropriately reduced. We used the Levitt-6 grouping, which is based on amino acids properties such as polarity and secondary structure propensity. As described in Section 2, polarity and secondary structure propensity seem to be important to carbohydrate binding. Because of the high frequency of cysteine in the positive dataset (Figure 2), we also used the Someya-7 grouping, an extension of Levitt-6 that treats cysteine as a separate group. This is based on the high frequency of cysteine of the positive dataset that is found by our amino acid frequency analysis (Figure 2). Some families of carbohydrate-binding proteins have cysteine-rich domains such as EG domains and LE domains which are found in many matrix molecules. Transmembrane C-type lectins also contain a cysteine-rich domain at the N-terminal side.

The ROC curves in Figure 3 indicate that the discrimination ability of the Someya-7 grouping method is better than that of Levitt-6 method and is comparable to that of the AA-20 grouping method. Someya-7 required less computation time than AA-20, taking 3 hours 7 minutes to predict against 185,003 sequences of HIPs (Release 5.0) in the H-InvDB [36], while AA-20 took 8 hours 55 minutes to complete. Although Someya-7 was faster, the TPR of AA-20 was higher than that of the Someya-7.

Figure 4 shows the performance for the genome-wide prediction against HIPs. As shown in this figure, the TPR obtained using the combined method was better than that obtained using the homology-based method. In the sequences predicted as positive by the combined method, the sequences with the keywords of “adhesion,” “matrix,” and “immunoglobulin-like” were found more frequently than those predicted by the SVM-based method alone or the homology-based method alone. In addition, the SVM-based method found 492 carbohydrate-binding proteins not found by the homology-based method. These included fibronectin type III domain containing protein 4 precursor, heparin-binding growth factor 1 precursor, fibronectin type III and ankyrin repeat domains protein, interleukin 1 receptor type II precursor, and galectin III. Precise evaluation, however, is not yet possible because the current version of H-InvDB is not fully annotated and might contain many carbohydrate-binding proteins that have not been annotated. The accuracy of the evaluation depends on the quality of the database annotation.

In this study we used clustering to remove the sequence redundancy of the positive dataset. Since the number of positive samples available was small, it was difficult to decrease the threshold value of clustering (i.e., to reduce the redundancy of the positive dataset). Thus, the threshold value had to be set at a level that would maintain the size of the positive dataset and assure its nonredundancy. In the genome-wide prediction for H-invDB, in which sequences were regarded as positive when they had enough homology with the sequences of the positive dataset, we showed that the SVM-based method could detect carbohydrate-binding proteins that could not be detected by the homology-based method. We believe that it will be even more useful when more positive samples become available. 

## 5. Conclusion

We have developed an SVM-based system for predicting carbohydrate-binding proteins from their sequences. This system is intended to provide information to support laboratory experimentation. As more data from work on high-throughput glycol proteomics becomes available and more knowledge is acquired, the reliability of our system's predictions should improve because SVM performance depends on the features extracted and the quality of the training dataset. We have also tested group encoding methods other than ones described in this paper and they did not achieve performance better than Someya-7 and Levitt-6. Many other grouping methods are, however, conceivable and the analysis of them will be part of the future work in this study. We also implemented the homology-based method and combined it with the SVM-based methods. Using these methods in genome-wide analysis of H-invDB, we showed that SVMs are useful for improving the accuracy of homology-based prediction. The prediction system using SVMs is now available on our Web site at (http://bolero.bi.a.u-tokyo.ac.jp:8201/Lectin-Predictor/.)

## Supplementary Material

The following table lists the carbohydrate-binding domains in the positive dataset found in NCBI Conserved Domains Database (CDD).

## Figures and Tables

**Table 1:** List of query commands applied for a sequence retrieval system (SRS) to create a positive dataset.

Subsets	Search conditions in SRS Query Language	Number of hits	Number of hits in the positive dataset
Subset 1 Lectin which are not enzymes	[libs = {swiss_prot trembl}-Description: lectin*] ∣ [libs-Keywords:Lectin*] ∣ [libs-Keywords:Chitin-binding*] ∣ [libs-Description:sugarbinding*] ! ([libs-Description:/^ EC/] ∣ [libs-Description:/ase$/]) ! [libs-Description: Putative*] ! [libs-Description:putative*] ! [libs-ProtExist: 4*] ! [libs-ProtExist: 5*] ! [libs-ProtExist: 3*] & [libs-SeqLength#30:]	2017	231

Subset 2 Lectin which are also enzymes	[libs = {swiss_prot trembl}-Description: lectin*] ∣ [libs-Keywords:Lectin*] ∣ [libs-Keywords:Chitin-binding*] ∣ [libs-Description: sugar-binding*] & ([libs-Description: *Peptidase*] ∣ [libs-Description: ligase*] ∣ [libs-Description: ribonuclease*] ∣ [libs-Description: *Protease*] ∣ [libs-Description: *Proteinase*] ∣ [libs-Keywords: *lipase*] ∣ [libs-Keywords: ribonuclease*] ∣ [libs-Keywords: *Protease*] ∣ [libs-Keywords: *Proteinase*] ∣ [libs-Keywords: *lipase*]) ! [libs-Description: Putative*] ! [libs-Description:putative*] ! [libs-ProtExist: 4*] ! [libs-ProtExist: 5*] ! [libs-ProtExist: 3*] & [libs-SeqLength#30:]	37	4

Subset 3 Other “Carbohydrate-binding” proteins	[libs = {swiss_prot trembl}-Keywords: Carbohydrate-binding*] ∣ [libs-Description:Carbohydrate-binding*] ! [libs-Description: CUT*] ! [libs-Description: Hydrolase*] ! [libs-Description:lyase*] ! [libs-Description: Putative*] ! [libs-Description:putative*] ! [libs-ProtExist: 4*] ! [libs-ProtExist: 5*] ! [libs-ProtExist: 3*] & [libs-SeqLength#30:]	16	15

Subset 4 Hyaluronic acid binding proteins	[libs = {swiss_prot trembl}-Description: Hyaluronate*] ∣[libs-Keywords:Hyaluronate*] ∣ [libs-Description: Hyaluronan*] ∣ [libs-Keywords:Hyaluronan*] ∣ [libs-Description: Hyaluronic*] ∣ [libs-Keywords:Hyaluronic*] ! [libs-Description: lyase*] ! [libs-Description: synthase*] & ([libs-Description: *link*] ∣ [libs-Description: *bind*] ∣ [libs-Description: *associate*] ∣ [libs-Description: *receptor*] ∣ [libs-Description: *mediate*] ∣ [libs-Keywords: *link*] ∣ [libs-Keywords: *bind*] ∣ [libs-Keywords: *associate*]) ! [libs-Description: Putative*] ! [libs-Description:putative*] ! [libs-ProtExist: 4*] ! [libs-ProtExist: 5*] ! [libs-ProtExist: 3*] & [libs-SeqLength#30:]	90	14

Subset 5 Heparin-binding proteins	[libs = {swiss_prot trembl}-Keywords: Heparin-binding*] ∣ [libs-Description:Heparin-binding*] ! [libs-Description: Putative*] ! [libs-Description:lyase*] ! [libs-Description:putative*] ! [libs-ProtExist: 4*] ! [libs-ProtExist: 5*] ! [libs-ProtExist: 3*] & [libs-SeqLength# 30:]	333	60

Subset 6 Interleukin which can bind to sugar-chains	[libs = {swiss_prot trembl}-ID: IL1A_*] ∣ [libs-ID: IL1B_*] ∣ [libs-ID: IL4_*] ∣ [libs-ID: IL1RA_*] ∣ [libs-ID: IL6_*] ∣ [libs-ID: IL3_*] ∣ [libs-ID: IL2_*] ! [libs-Description: Putative*] ! [libs-Description:putative*] ! [libs-ProtExist: 4*] ! [libs-ProtExist: 5*] ! [libs-ProtExist: 3*] & [libs-SeqLength#30:]	154	7

Subset 7 FimH adhesion of type 1 pili	[libs = {swiss_prot trembl}-Description: FimH*] ∣ [libs-Description: Neuraminyllactose-binding*] ∣ [libs-Description: S-fimbrial adhesin*] ! [libs-Description: Putative*] ! [libs-Description:putative*] ! [libs-ProtExist: 4*] ! [libs-ProtExist: 5*] ! [libs-ProtExist: 3*] & [libs-SeqLength#30:])	1	1

Subset 8 F-box only protein which can bind to sugar-chains	[libs = {swiss_prot trembl}-ID: FBX27_HUMAN*] ∣ [libs-ID: FBX6_HUMAN*]	2	1

Subset 9 Agrin. Tenascin-C Phospholipase A2 inhibitor subunit A Neurexin	[libs = {swiss_prot trembl}-ID: AGRIN_HUMAN] ∣ [libs-ID: PLIA_TRIFL] ∣ [libs-Description: Tenascin-C] ∣ [libs-ID: NRX1A_HUMAN*]	13	8

Subset 10 Chitin-binding proteins	[libs = {swiss_prot trembl}-Description: cbp-1] ! [libs-Description: Centromere* ] ! [libs-Description: EC*] ! [libs-Description: synthase*] ! [libs-Description: Putative*] ! [libs-Description:putative*] ! [libs-ProtExist: 4*] ! [libs-ProtExist: 5*] ! [libs-ProtExist: 3*] & [libs-SeqLength#30:]	4	4

**Table:** (a)

	Nonpolar	Polar
*α*-Helix	A, C, L, M	E, H, K, Q, R
*β*-Strand	F, I, V, W, Y	T
Turn	G, P	D, N, S

**Table:** (b)

	Nonpolar	Polar
*α*-Helix	A, L, M	E, H, K, Q, R
*β*-Strand	F, I, V, W, Y	T
Turn	G, P	D, N, S
Cysteine	C	

**Table 3:** List of Performance measures.

	AA-20	Levitt-6	Someya-7
ACC	0.87	0.83	0.84

TPR	0.83	0.77	0.80
FPR	0.09	0.11	0.11
MCC	0.74	0.67	0.70

AUC	0.929	0.890	0.918

The performance measures are obtained through the leave-one-out method with a classification threshold (decision value) of *θ* = 0 and the AUCs of AA-20, Levitt-6, and Someya-7 grouping methods.

Abbreviations: ACC: accuracy, TPR: true positive rate, FPR: false positive rate, MCC: Matthews correlation coefficient, and AUC: area under the ROC curve.

## References

[1] Sharon N, Lis H (2003). *Lectins*.

[2] Vapnik V (1998). *Statistical Learning Theory*.

[3] Wang LH, Liu J, Li YF, Zhou HB (2004). Predicting protein secondary structure by a support vector machine based on a new coding scheme. *Genome Informatics Series*.

[4] Ward JJ, Sodhi JS, McGuffin LJ, Buxton BF, Jones DT (2004). Prediction and functional analysis of native disorder in proteins from the three kingdoms of life. *Journal of Molecular Biology*.

[5] Ding CHQ, Dubchak I (2001). Multi-class protein fold recognition using support vector machines and neural networks. *Bioinformatics*.

[6] Shionyu-Mitsuyama C, Shirai T, Ishida H, Yamane T (2003). An empirical approach for structure-based prediction of carbohydrate-binding sites on proteins. *Protein Engineering*.

[7] Malik A, Ahmad S (2007). Sequence and structural features of carbohydrate binding in proteins and assessment of predictability using a neural network. *BMC Structural Biology*.

[8] Leslie C, Eskin E, Noble WS The spectrum kernel: a string kernel for SVM protein classification.

[9] Smith AK, Cheung K-H, Yip KY (2007). The Universal Protein Resource (UniProt). *Nucleic Acids Research*.

[10] Endo T (2003). Finding of O-mannosyl glycan in mammals and congenital muscular dystrophies due to glycosylation defects. *Yakugaku Zasshi*.

[11] Kamiya Y, Yamaguchi Y, Takahashi M (2005). Sugar-binding properties of VIP36, an intracellular animal lectin operating as a cargo receptor. *The Journal of Biological Chemistry*.

[12] Sharon N (2007). Lectins: carbohydrate-specific reagents and biological recognition molecules. *The Journal of Biological Chemistry*.

[13] Pyz E, Marshall ASJ, Gordon S, Brown GD (2006). C-type lectin-like receptors on myeloid cells. *Annals of Medicine*.

[14] Deepa SS, Umehara Y, Higashiyama S, Itoh N, Sugahara K (2002). Specific molecular interactions of oversulfated chondroitin sulfate E with various heparin-binding growth factors: implications as a physiological binding partner in the brain and other tissues. *The Journal of Biological Chemistry*.

[15] Aricescu AR, McKinnell IW, Halfter W, Stoker AW (2002). Heparan sulfate proteoglycans are ligands for receptor protein tyrosine phosphatase *σ*. *Molecular and Cellular Biology*.

[16] Kallapur SG, Akeson RA (1992). The neural cell adhesion molecule (NCAM) heparin binding domain binds to cell surface heparan sulfate proteoglycans. *Journal of Neuroscience Research*.

[17] Mohan PS, Chou DKH, Jungalwala FB (1990). Sulfoglucuronyl glycolipids bind laminin. *Journal of Neurochemistry*.

[18] Cole GJ, Akeson R (1989). Identification of a heparin binding domain of the neural cell adhesion molecule N-CAM using synthetic peptides. *Neuron*.

[19] Cebo C, Vergoten G, Zanetta J-P (2002). Lectin activities of cytokines: functions and putative carbohydrate-recognition domains. *Biochimica et Biophysica Acta*.

[20] Vergoten G, Zanetta J-P (2007). Structural differences between the putative carbohydrate-recognition domains of human IL-1 alpha, IL-1 beta and IL-1 receptor antagonist obtained by in silico modeling. *Glycoconjugate Journal*.

[21] Parkkinen J, Raulo E, Merenmies J (1993). Amphoterin, the 30-kDa protein in a family of HMG1-type polypeptides. Enhanced expression in transformed cells, leading edge localization, and interactions with plasminogen activation. *The Journal of Biological Chemistry*.

[22] Apweiler R, Gateau A, Contrino S Protein sequence annotation in the genome era: the annotation concept of SWISS-PROT+TREMBL.

[23] Apweiler R (2001). Functional information in SWISS-PROT: the basis for large-scale characterisation of protein sequences. *Briefings in Bioinformatics*.

[24] Schäffer AA, Wolf YI, Ponting CP, Koonin EV, Aravind L, Altschul SF (1999). IMPALA: matching a protein sequence against a collection of PSI-BLAST-constructed position-specific score matrices. *Bioinformatics*.

[25] Altschul SF, Madden TL, Schäffer AA (1997). Gapped BLAST and PSI-BLAST: a new generation of protein database search programs. *Nucleic Acids Research*.

[26] Thompson JD, Higgins DG, Gibson TJ (1994). CLUSTAL W: improving the sensitivity of progressive multiple sequence alignment through sequence weighting, position-specific gap penalties and weight matrix choice. *Nucleic Acids Research*.

[27] Marchler-Bauer A, Panchenko AR, Shoemarker BA, Thiessen PA, Geer LY, Bryant SH (2002). CDD: a database of conserved domain alignments with links to domain three-dimensional structure. *Nucleic Acids Research*.

[28] Levitt M (1978). Conformational preferences of amino acids in globular proteins. *Biochemistry*.

[29] Rini JM (1995). Lectin structure. *Annual Review of Biophysics and Biomolecular Structure*.

[30] Chandra NR, Prabu MM, Suguna K, Vijayan M (2001). Structural similarity and functional diversity in proteins containing the legume lectin fold. *Protein Engineering*.

[31] Wright LM, Van Damme EJM, Barre A (1999). Isolation, characterization, molecular cloning and molecular modelling of two lectins of different specificities from bluebell (Scilla campanulata) bulbs. *Biochemical Journal*.

[32] Hamelryck TW, Loris R, Bouckaert J, Wyns L (1998). Structural features of the legume lectins. *Trends in Glycoscience and Glycotechnology*.

[33] Hester G, Kaku H, Goldstein IJ, Wright CS (1995). Structure of mannose-specific snowdrop (Galanthus nivalis) lectin is representative of a new plant lectin family. *Nature Structural Biology*.

[34] Torian BE, Flores BM, Stroeher VL, Hagen FS, Stamm WE (1990). cDNA sequence analysis of a 29-kDa cysteine-rich surface antigen of pathogenic Entamoeba histolytica. *Proceedings of the National Academy of Sciences of the United States of America*.

[35] Fawcett T (2004). ROC graphs: notes and practical considerations for researchers. *HP Labs Technical Reports*.

[36] Genome Information Integration Project and H-Invitational 2 (2008). The H-Invitational Database (H-InvDB), a comprehensive annotation resource for human genes and transcripts. *Nucleic Acids Research*.

