# Supplementary material for: Prediction of Carbohydrate-Binding Proteins from Sequences Using Support Vector Machines
Source: Adv Bioinformatics. 2010 Sep 27;2010:289301. doi: 10.1155/2010/289301 (PMC2948896; doi:10.1155/2010/289301)
Supplement: Supplementary file 1 — The following table lists the carbohydrate-binding domains in the positive dataset found in NCBI Conserved Domains Database (CDD). [file 289301.f1.pdf]

CDD ID	Number of hits	Description of domains
smart00034	16	C-type lectin (CTL) or carbohydrate-recognition domain (CRD)
cd03593	13	CLECT_NK_receptors_like
cd03590	11	CLECT_DC-SIGN_like
cd00037	10	CLECT: C-type lectin (CTL)/C-type lectin-like (CTLD) domain
cd00070	7	Galectin/galactose-binding lectin
cd03588	5	CLECT_CSPGs
pfam00337	5	Galactoside-binding lectin
smart00494	5	Chitin-binding domain type 2
pfam00139	4	Legume lectin domain
pfam01419	4	Jacalin-like lectin domain
pfam03388	4	Legume-like lectin family
pfam00652	4	Ricin-type beta-trefoil lectin domain
cd03589	4	CLECT_CEL-1_like
cd03592	3	CLECT_selectins_like
cd00161	3	Ricin-type beta-trefoil
cd00087	3	Fibrinogen-related domains (FReDs)
COG1196	3	Chromosome segregation ATPases (Cell division and chromosome partitioning)
pfam00059	3	Lectin C-type domain
cd00190	3	Trypsin-like serine protease
smart00276	3	Galectin
cd03591	3	CLECT_collectin_like
cd00028	2	Bulb-type mannose-specific lectin
pfam00167	2	Fibroblast growth factor
pfam00262	2	Calreticulin family
smart00125	2	Interleukin-1 homologues
smart00020	2	Trypsin-like serine protease
pfam07686	2	Immunoglobulin V-set domain
pfam01442	2	Apolipoprotein A1/A4/E domain
cd03516	2	This domain is a hyaluronan (HA)-binding domain
smart00636	2	Glyco_18 domain
smart00607	2	eel-Fucolectin Tachylectin-4 Pentaxrin-1 Domain
pfam02059	2	Interleukin-3
pfam00354	2	Pentaxin family
pfam04774	2	Hyaluronan / mRNA binding family
cd04273	2	Zinc-dependent metalloprotease
cd03600	2	CLECT_thrombomodulin_like
pfam07679	2	Immunoglobulin I-set domain
pfam08205	2	CD80-like C2-set immunoglobulin domain
cd03515	2	ink_domain_TSG_6_like
pfam01179	2	Copper amine oxidase
smart00458	2	Ricin-type beta-trefoil
smart00706	2	Beta propeller repeats in Physarum polycephalum tectonins
cd03594	2	CLECT_REG-1_like
cd00033	2	Domain abundant in complement control proteins

cd00035	2	Chitin binding domain
pfam07938	2	Fungal fucose-specific lectin
pfam00386	2	C1q domain
pfam00041	2	Fibronectin type III domain
pfam01453	2	D-mannose binding lectin
pfam00715	1	Interleukin 2
pfam00489	1	Interleukin-6/G-CSF/MGF family
pfam00727	1	Interleukin 4
pfam02140	1	Galactose binding lectin domain
pfam00151	1	Lipase
smart00141	1	Platelet-derived and vascular endothelial growth factors (PDGF)
cd00931	1	Immunoglobulin domain cell adhesion molecule (cam) subfamily
smart00193	1	Pleiotrophin / midkine family
smart00199	1	Intercrine alpha family (small cytokine C-X-C) (chemokine CXC)
pfam02354	1	Latrophilin Cytoplasmic C-terminal region
smart00058	1	Fibronectin type 1 domain
pfam00007	1	Cystine-knot domain
cd00273	1	Chemokine_CXC
pfam05735	1	Thrombospondin C-terminal region
pfam02177	1	Amyloid A4 extracellular domain
cd00041	1	CUB domain
pfam04881	1	Adenovirus GP19K
smart00409	1	Immunoglobulin
pfam09014	1	Beta-2-glycoprotein-1 fifth domain
cd01328	1	Follistatin-like SPARC (secreted protein)
pfam01776	1	Ribosomal L22e protein family
pfam05927	1	Penaeidin
pfam00147	1	Fibrinogen beta and gamma chains
cd03597	1	CLECT_attractin_like
cd02045	1	Antithrombin is a serine proteinase inhibitor (serpin) which controls the process of coagulation
cd03595	1	CLECT_chondrolectin_like
cd03596	1	CLECT_tetranectin_like
pfam00431	1	CUB domain
cd03601	1	CLECT_TC14_like
smart00038	1	Fibrillar collagens C-terminal domain
pfam00030	1	Beta/Gamma crystallin
smart00200	1	Domain found in sea urchin sperm protein
smart00202	1	Scavenger receptor Cys-rich
smart00198	1	SCP / Tpx-1 / Ag5 / PR-1 / Sc7 family of extracellular domains
cd02047	1	Heparin cofactor II (HCII) inhibits thrombin
cd00094	1	Hemopexin-like repeats
cd00057	1	Coagulation factor 5/8 C-terminal domain
pfam00855	1	PWWP domain

smart00281	1	Laminin B domain
pfam06668	1	Inter-alpha-trypsin inhibitor heavy chain C-terminus
smart00219	1	Tyrosine kinase
pfam00031	1	Cystatin domain
pfam07915	1	Glucosidase II beta subunit-like protein
smart00159	1	Pentraxin / C-reactive protein / pentaxin family
cd02051	1	Plasminogen activator inhibitor-1_like
pfam06401	1	Alpha-2-macroglobulin RAP
cd01100	1	Subfamily of PAN/APPLE-like domains
pfam07367	1	Fungal fruit body lectin
pfam09458	1	H-type lectin domain
smart00638	1	Lipoprotein N-terminal Domain
smart00408	1	Immunoglobulin C-2 Type
pfam02469	1	Fasciclin domain
smart00006	1	amyloid A4
cd03599	1	CLECT_DGCR2_like: C-type lectin-like domain (CTLD) of the type found in DGCR2
pfam00100	1	Zona pellucida-like domain
cd03576	1	NTR domain
pfam08531	1	Alpha-L-rhamnosidase N-terminal domain
pfam00191	1	Annexin
pfam04300	1	F-box associated region
pfam03498	1	Cytolethal distending toxin A/C family
smart00059	1	Fibronectin type 2 domain
pfam00379	1	Insect cuticle protein
pfam07472	1	Fucose-binding lectin II (PA-IIL)
smart00216	1	von Willebrand factor (vWF) type D domain
pfam06010	1	Domain of Unknown Function (DUF906)
pfam03146	1	Agrin NtA domain
smart00282	1	Laminin G domain
pfam00839	1	Cysteine rich repeat
cd00078	1	HECT domain
cd03520	1	Link_domain_CSPGs_modules_2_4
pfam07968	1	Leukocidin/Hemolysin toxin family
cd01477	1	VWA F09G8
cd01471	1	Micronemal proteins
COG5126	1	Ca2+-binding protein (EF-Hand superfamily) [Signal transduction mechanisms / Cytoskeleton / Cell division and chromosome partitioning / General function prediction only]
cd04280	1	Zinc-dependent metalloprotease
smart00360	1	RNA recognition motif
cd01450	1	Von Willebrand factor type A (vWA) domain was originally found in the blood coagulation protein von Willebrand factor (vWF)
pfam01607	1	Chitin binding Peritrophin-A domain
cd00118	1	Lysin domain
cd00083	1	Helix-loop-helix domain

pfam09222	1	Fimbrial adhesin F17-AG
cd03602	1	CLECT_1
pfam09160	1	FimH
pfam08277	1	PAN-like domain
pfam02330	1	Mitochondrial glycoprotein
cd00241	1	Cellobiose dehydrogenase (CellobioseDH)
pfam00282	1	Pyridoxal-dependent decarboxylase conserved domain
pfam00080	1	Copper/zinc superoxide dismutase (SODC)
pfam06473	1	FGF binding protein 1 (FGF-BP1)
smart00637	1	CBD_II domain
smart00236	1	Fungal-type cellulose-binding domain
cd00062	1	Fibronectin Type II domain
cd00054	1	Calcium-binding EGF-like domain
pfam05963	1	Cytomegalovirus US3 protein
COG2217	1	Cation transport ATPase [Inorganic ion transport and metabolism]
pfam01779	1	Ribosomal L29e protein family
pfam00068	1	Phospholipase A2
pfam00074	1	Pancreatic ribonuclease
pfam07828	1	PA-IL-like protein
pfam09259	1	Fungal immunomodulatory protein Fve
cd00061	1	Fibronectin type 1 domain

Table S1 Domains found in the positive dataset.
